# Supplementary material for: The impact of properly diagnosed sarcopenia on postoperative outcomes after gastrointestinal surgery: A systematic review and meta-analysis
Source: PLoS One. 2020 Aug 21;15(8):e0237740. doi: 10.1371/journal.pone.0237740 (PMC7446889; doi:10.1371/journal.pone.0237740)

**THE IMPACT OF PROPERLY DIAGNOSED SARCOPENIA ON POSTOPERATIVE OUTCOMES AFTER GASTROINTESTINAL SURGERY: A SYSTEMATIC REVIEW AND META-ANALYSIS**

**Authors**

Leonardo Zumerkorn Pipek ^1^, Carlos Guilherme Baptista ^3^, Rafaela Farias Vidigal Nascimento ^2^, João Victor Taba ^1^, Milena Oliveira Suzuki ^1^, Fernanda Sayuri do Nascimento ^1^, Diego Ramos Martines ^1^, Fernanda Nii ^1^, Leandro Ryuchi Iuamoto ^3^, Luiz Augusto Carneiro-D’Albuquerque ^3^, Alberto Meyer^3*^, Wellington Andraus ^3^

SUPPLEMENT MATERIAL

**S1 Table**

**Comparison between sarcopenic and non-sarcopenic groups regarding comorbidities and cancer stage**


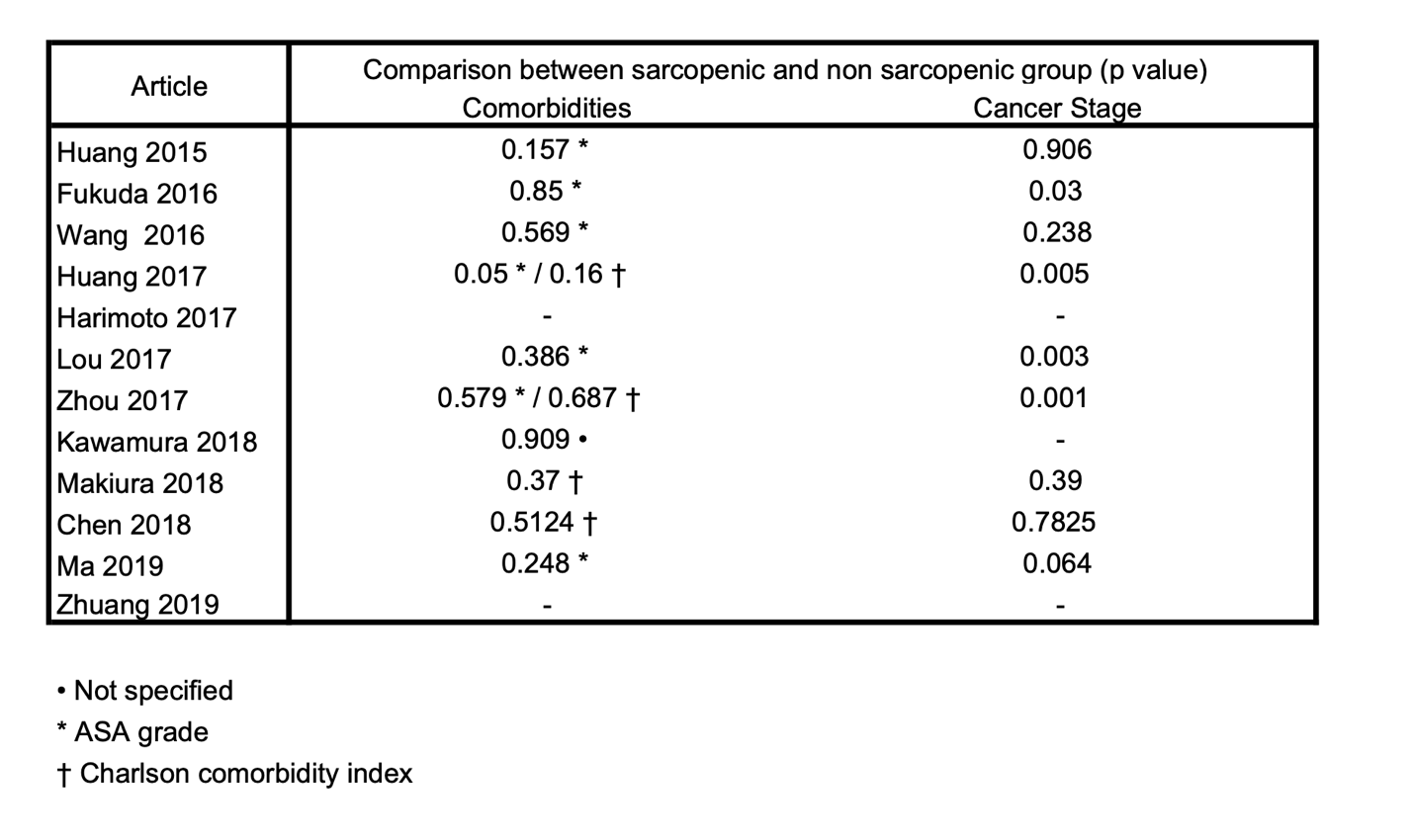

Supplement: S1 File — (DOCX) [file pone.0237740.s001.docx]
