# Supplementary material for: The impact of properly diagnosed sarcopenia on postoperative outcomes after gastrointestinal surgery: A systematic review and meta-analysis
Source: PLoS One. 2020 Aug 21;15(8):e0237740. doi: 10.1371/journal.pone.0237740 (PMC7446889; doi:10.1371/journal.pone.0237740)
Supplement: Attachment — Submitted filename: Response to Reviewers.docx [file pone.0237740.s003.docx]

Dear reviewers,

We really appreciate all the comments, we believe our text is now substantially better.

#1: This is an interesting systematic review and meta-analysis on the impact of sarcopenia on the postoperative outcomes following gastrointestinal surgeries. The clinical question is well posed and the topic is timely an appropriate. Statistics are well performed. PRISMA guidelines are strictly respected and protocol registration on an international database makes the analysis of high quality. Conclusions are supported by the results. I have some comments that could improve the manuscript.
There is an important manuscript published in the literature a couple of years ago concerning Sarcopenia as a predictor of morbidity in gastrointestinal surgical oncology. This is a systematic review and meta-analysis (Simonsen et al. Ann. Surg. 2018). I don't know why this article is not even mentioned in your manuscript. Was this missed? You definetely have to comment this manuscript in your paper as it is a major one.

Thank you for pointing out this article. Indeed, this was very valuable, and we added to our discussion. (Reference 52).

Yours have some significant improvements i believe so please show the reader that it is worth it to have a new one on the topic.

We have now emphasized this in our title, abstract and in the body of the text.
You have correctly mentioned that because of the absolute prevalence of Eastern studies in your analysis, your results could not be reproducible among Westerners. I think that it is worth mentioning that another issue is the use of different cutoffs among studies as it is clear from your Tables. Indeed, some authors use recognized cutoffs to divide sarcopenic and non sarcopenic patients while others use ROC curves on their populations. Both methods have limitations of course and therefore this needs to be mentioned as a limitation in your study. We currently don't have an exact cutoff to define sarcopenic patients homogenously.

We totally agree with you and added this limitation to our discussion.
Finally, of course the real novelty here is that you consider the 1 and only correct definition of sarcopenia which is made up of both muscle mass and muscle strength . The authors should emphasize this in their study. In my opinion also in the title. If you leave your title as it is, you might get the reader confused. Indeed, when i read your title to review i thought that i was about t read something with no novelty (Very similar to the one from Simonsen et al.). But indeed you have something new and important to tell. Please consider giving your title some more appeal.

Thank you very much for this insight, we changed our title to show the novelty of our study.

Reviewer #2: This is a nice systematic review and meta-analysis to address the role of sarcopenia in the postoperative complications of gastrointestinal surgery. Authors have found that sarcopenia was significantly associated with an increase in late postoperative complications, as well as an increase in the number of postoperative hospital readmissions after gastrointestinal surgery. Thus, it is important to assess sarcopenia and provide appropriate interventions before surgery to reduce its impact on the patient's health. I think that this is a well-conducted systematic review according to the PRISM statement. However, liver transplantation should not be included in gastrointestinal surgery and the data should be removed from the analysis.

We removed this study (Harimoto et al. 2017) and changed all figures and tables. The final results of our meta analyses barely changed. We didn’t have to change any conclusion about our analyses.
Minor point.
References 4 and 23 are overlapped.

We corrected this and also included new references that were suggested (ref 44 and 52).
